# The relationship between grit, resilience and physical activity: a systematic review

**DOI:** 10.3389/fspor.2025.1563382

**Published:** 2025-07-07

**Authors:** Joel Martin, Ashley Hromyak, Megan Sax van der Weyden, Ali A. Weinstein, Ali Boolani

**Affiliations:** ^1^ Sports Medicine Assessment Research & Testing (SMART) Laboratory, School of Kinesiology, George Mason University, Manassas, VA, United States; ^2^ Center for the Advancement of Well-Being, George Mason University, Fairfax, VA, United States; ^3^ Department of Global and Community Health, College of Public Health, George Mason University, Fairfax, VA, United States; ^4^ Human Performance and Nutrition Research Institute, The Innovation Foundation, Oklahoma State University, Stillwater, OK, United States; ^5^ Department of Pharmacology and Physiology, Oklahoma State University College of Medicine, Tulsa, OK, United States; ^6^ Department of Mechanical and Aeronautical Engineering, Clarkson University, Potsdam, NY, United States

**Keywords:** physical activity, exercise adherence, health, grit, resilience, health promotion

## Abstract

**Introduction:**

At present only about half of Americans meet the recommended physical activity (PA) guidelines. Theoretically personality traits, encompassed by grit and resilience, should be beneficial to overcome common barriers to PA participation. To systematically review synthesized literature regarding the relationship between grit, resilience, and PA outcomes.

**Methods:**

The search methodology adhered to the Preferred Reporting Items for Systematic Review and Meta-Analyses (PRISMA) guidelines. Study eligibility criteria included peer-reviewed studies with healthy adult participants, where a reported relationship between PA and either grit or resilience existed. Study quality was evaluated with the Appraisal Tool for Cross-Sectional Studies (AXIS) and a qualitative synthesis was performed.

**Results:**

A total of 33 studies involving diverse participants (*n* = 37,370) across age, sex, culture, education, and PA outcomes met the inclusion criteria. The methodological quality of studies was rated as good on average. Most of the studies found positive relationships between grit, resilience, and PA outcomes, such as adherence, intensity, and performance in competitive settings.

**Conclusion:**

Cumulatively, the findings suggest that personality traits of grit and resilience play a significant role in supporting PA engagement, with individuals exhibiting higher levels being more likely to participate in regular PA and achieve better performance outcomes. Given that most studies employed cross-sectional designs, future research is needed to elucidate whether a causal relationship exists between grit, resilience, and PA. These findings may have practical applications for designing interventions aimed at fostering these traits to enhance PA adherence and overall health.

**Systematic Review Registration:**

https://www.crd.york.ac.uk/PROSPERO/view/CRD42022370061, identifier CRD42022370061.

## 1 Introduction

Physical activity (PA) plays an essential role in promoting overall health and well-being throughout the lifespan (1). PA is defined as any bodily movement that results in energy expenditure, which is an umbrella term for various activities including exercise and sport (2). Regular engagement in PA has been consistently linked to numerous physical and mental health benefits, including reduced risk of chronic diseases, improved cardiovascular fitness, enhanced cognitive function, better mental health, and increased longevity (1). Despite the current PA guidelines, recommending at least 150 min of moderate to vigorous intensity per week (3), only half of Americans meet these guidelines (4).

Numerous studies conducted across diverse countries and communities have identified environmental barriers, such as neighborhood design and perceived safety, as significant influences on adults' PA levels (5–7). A common barrier is the lack of access to convenient facilities or designated spaces for exercise, whereas a well-designed built environment can serve as a strong facilitator of PA (8). Perceived neighborhood safety also plays a critical role in PA engagement. Individuals who live in areas with high levels of crime or who do not feel safe outdoors are less likely to engage in outdoor activities such as walking, jogging, or recreational play (7). Additionally, time constraints are consistently reported as a universal barrier across adult populations, limiting regular engagement in PA (5). These environmental barriers interact with individual-level determinants and contribute to disparities in PA across socioeconomic and geographic groups (6, 9). Among these individual-level factors, personality traits have received growing attention. Personality traits are defined as enduring patterns of thought, emotion, and behavior that remain relatively stable over time and across contexts (10). These traits influence how individuals perceive barriers, cope with setbacks, and sustain motivation, making them highly relevant to understanding PA behaviors (11). Accordingly, a growing body of evidence suggests that personality traits (12, 13) may positively influence PA participation (14–16).

One such personality trait, grit, which is defined as passion and perseverance towards long-term goals despite failures (17), may be beneficial for engaging in PA. The construct of grit, introduced in 2007 as a trait encompassing perseverance and consistency of long-term interests, has been found to be a robust predictor of accomplishments in challenging domains, often surpassing conventional measures of talent (17). Subsequent research indicates that individuals with higher levels of grit demonstrate success across diverse domains, including academics, sports, and professional settings (18, 19). Grit plays a vital role in sustaining effort, enabling individuals to navigate obstacles and setbacks that might otherwise impede the pursuit of their goals (20). Self-report measures, such as the 12-item Grit-O scale developed by Duckworth and colleagues (17), serves as a common tool in literature to gauge grit (20). This scale provides an overall grit score and sub-component scores for perseverance of effort and consistency of interest.

**Figure 1:**
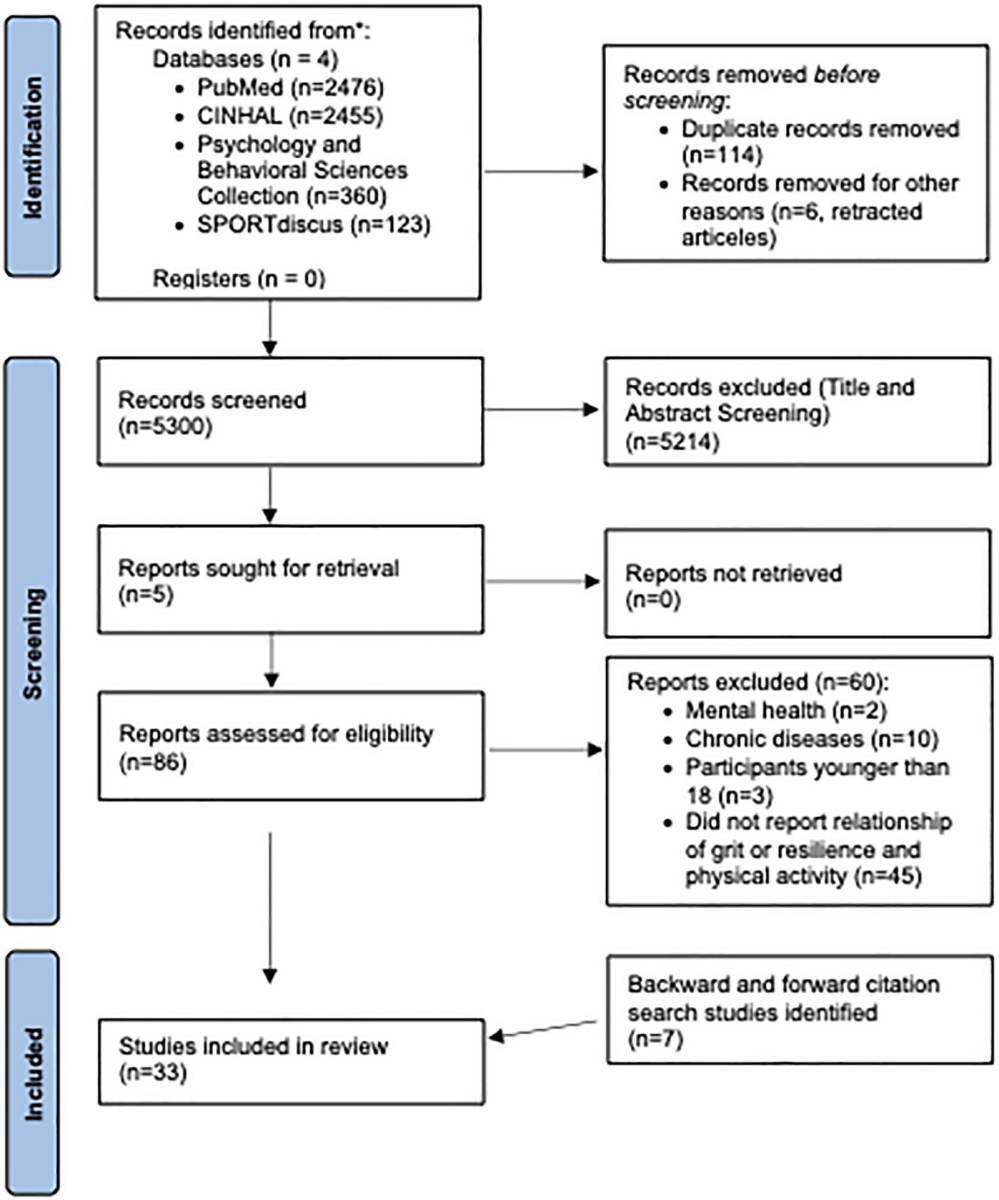
Conceptual “invest and accrue” model illustrating how the perseverance of effort component of grit can support sustained physical activity engagement. Gritty individuals are hypothesized to invest effort into physical activity behaviors despite short-term challenges, accruing health benefits over time that reinforce sustained motivation. PA, physical activity.

The perseverance of effort component of grit is highly correlated with conscientiousness (21), a personality trait where individuals invest in behaviors that allows for future successes (22). This “invest and accrue” model (Figure 1) employed by conscientious people also finds that conscientious individuals invest in their physical health by adopting healthy behaviors (22). These findings imply that gritty individuals, who are high on the perseverance aspect of grit, may also employ a similar “invest and accrue” model of health behavior, thus being more physically active compared to their less “gritty” counterparts. These individuals may be more likely to overcome obstacles such as time constraints, low self-confidence, competing priorities, and difficulty overcoming setbacks (5, 13, 23, 24). Consequently, higher grit may foster sustained engagement in PA, driven by the perceived benefits towards future health goals (19).

The second component of grit, consistency of interest, also emerges as a potential influencer of PA behaviors across the lifespan, as discerned from studies demonstrating that individuals reporting higher scores on this aspect tend to sustain similar interests for prolonged durations (25). This inclination suggests that those scoring higher on consistency of interest may exhibit sustained levels of PA over extended periods, potentially leading to higher fitness levels that facilitate engagement in more prolonged and intense exercise regimens, such as performing moderate to vigorous PA rather than light PA (26). Research investigating the interplay between grit and PA corroborates these observations, highlighting that both perseverance and consistency of interest are positively correlated with PA intensity (14, 27). Thus, given the detrimental health consequences associated with low levels of PA (e.g., obesity) and the plausible relationship between PA engagement and grit, researchers have increasingly examined the relationship between grit and PA measures (14, 16, 28, 29).

A construct closely related to grit is resilience (30). Although grit and resilience are often discussed together, sometimes used interchangeably in the literature (31) and have been combined in studies (32), they represent distinct psychological constructs with unique theoretical foundations and behavioral implications (25, 33). Grit is conceptualized as a personality trait reflecting sustained passion and perseverance toward long-term goals, characterized by continued effort despite failure, stagnation, or adversity (25). In contrast, resilience refers to a dynamic, context-dependent process of positive adaptation in the face of stress, adversity, or trauma (33). Resilient individuals demonstrate an ability to recover from setbacks and maintain psychological functioning, often adapting strategies and learning from challenging experiences (33). In other words, while both constructs involve persistence through difficulty, grit emphasizes unwavering goal-directed effort over time, whereas resilience centers on flexible coping and psychological recovery (34). This distinction underscores the need to examine grit and resilience as related but different contributors to behavior and motivation.

Despite their conceptual differences, grit and resilience may function in a complementary manner to support long-term goal attainment, such as sustained PA engagement. In theory grit provides the foundation for consistent, effortful pursuit of valued goals, even in the absence of immediate rewards or progress (25). Resilience, by contrast, facilitates adaptive functioning when disruptions or stressors threaten progress toward those goals (33). For example, in the context of PA, a gritty individual may persist with an exercise routine when faced with environmental barriers, during periods of low motivation or plateaus in performance, whereas a resilient individual may adapt the routine in response to injury or life stress, maintaining engagement through flexibility. *n* this way, resilience may enhance the sustainability of gritty behaviors by facilitating coping and recovery, while grit ensures directional consistency and persistence. Moreover, resilience may develop as an outcome of gritty behavior, as overcoming obstacles may strengthen adaptive capacities over time (35). Empirical research has demonstrated a moderate positive correlation between grit and resilience (36), with a stronger relationship between resiliency and the perseverance of effort component of grit (30, 37). However, resilience extends beyond perseverance, encompassing a broader range of coping strategies and adjustment processes (33). Therefore, when examining the association between grit and PA, resiliency should also be considered as it adds a dynamic and changeable process that is complementary to the more static grit personality trait (30, 32, 36, 37).

As with other personality traits, grit and resilience are not fixed and may change across the lifespan as a result of life experiences and contextual influences (38). For example, evidence suggests that grit varies across age (39, 40) and sex (39), with older individuals and women reporting significantly higher grit. Additionally, higher educational attainment has been positively associated with both grit (41) and PA participation (42). Beyond individual differences, grit and resilience are also influenced by cultural context (20, 43, 44). While these constructs are frequently studied within Western, individualistic societies, their meanings and manifestations may differ in collectivist cultures. In individualist societies, grit typically reflects autonomous persistence toward self-determined goals and emphasizes consistency of personal interest (25). Resilience in these contexts is often framed as an internal capacity for emotional regulation and recovery (33). In contrast, collectivist cultures may emphasize perseverance of effort in support of communal or family-oriented goals, rather than consistent personal interests, and may foster resilience through relational networks and communal coping strategies (20, 43, 44). For instance, individuals in collectivist societies may demonstrate high levels of perseverance through a sense of duty, even if their personal goals shift, and may rely more heavily on social support as a key resilience mechanism (43). Similarly, resilience may be expressed through reliance on social support and group cohesion (45). The cultural distinctions highlight the need to interpret grit and resilience in context and may influence how these constructs relate to PA behaviors across diverse populations (43). Taken together, sociodemographic and cultural factors may moderate the association between grit, resilience, and PA.

The significance of grit and resiliency for promoting positive PA behaviors is supported by a growing body of literature (14, 16, 46). Given the health issues associated with insufficient PA across the adult lifespan, comprehending the potential interactions between “grit” and “resilience” in supporting PA outcomes holds considerable value. Hence, the purpose of the systematic review was to synthesize the findings from studies examining the relationship between grit and resilience with PA. Within the overarching objective of the review, relationships with aspects of PA, such as adherence, PA intensity and engagement in various PA domains across the adult lifespan, were more specific relationships of interest.

## 2 Methods

The systematic literature search was conducted in accordance with the Preferred Reporting Items for Systematic Reviews and Meta-Analyses (PRISMA) guidelines (47) and the systematic review was registered in the International Prospective Register of Systematic Reviews (PROSPERO; registration #: CRD42022370061).

### 2.1 Eligibility criteria

The database search was confined to studies published in the English language peer-reviewed journals that met the following inclusion criteria: (a) participants were least 18 years of age, (b) published in the last 30 years, (c) peer reviewed, (d) investigated the quantitative relationship between grit, or resilience, and a measure of PA, (e) English language or translated, and (f) full text. The exclusion criteria applied during the search were: (a) articles that reported case studies, (b) abstracts only, and (c) studies that involved subjects with chronic disease and diagnosed mental health conditions who would not be considered healthy adults. For the purposes of the review healthy adults was considered free of disease or conditions that could interfere with their ability to engage in physical activity or complete grit/resilience questionnaires.

### 2.2 Search strategy

To obtain relevant literature, databases were searched using search terms relevant to the topic in October 2023. The following Boolean search syntax was used: (grit OR perseverance OR resilience) AND (physical activity OR exercise OR fitness OR physical exercise OR sport). Search terms were decided based on keywords retrieved from the reference pages of relevant articles on the topic and pilot testing. The search terms were applied to the search strategy for four databases: PubMed (Medline), CINHAL, Sport Discus, and Psychology and Behavioral Sciences Collection. Filters were applied in each database search, if available. If the filters were not available, investigators performed manual screening of titles and abstracts. The eligibility criteria were applied to the full-text articles not excluded during the screening of titles and abstracts to select the final number of studies to be included in the literature review. The reference list of each included article was used to perform a backward search for any additional articles that potentially would fit the search criteria. Forward citation tracking of the studies meeting the inclusion criteria was also performed. To reduce the potential errors the search was conducted independently by 2 of the authors (A.H. and J.M.). Any discrepancies were resolved by the authors, then a third author (M.S.) if needed.

### 2.3 Risk of bias and study quality evaluation

The risk of bias and quality of each study was independently assessed by 2 of the authors (A.H. and J.M), and agreement was mutually determined for any observed discrepancies. Given that 30 of 32 studies used a cross-sectional design, the study quality was evaluated by use of the 20-point Appraisal Tool for Cross-Sectional Studies (AXIS), which has been shown to be a valid measure of the methodological quality of cross-sectional studies (48). The responses using the AXIS tool are “yes,” “no,” or “don’t know,” and a numerical value was assigned for each response. An answer of “yes” was assigned a value of 1, an answer of “no” and “don’t know” was assigned a value of 0. Any responses that did not apply were given a response on N/A and was subtracted from the total of the 20-points. A total score was computed as the total score divided by the total number of possible points for each study and reported as a percentage. To interpret AXIS total scores, we categorized studies as high, moderate and low quality if they achieved ≥80%, 60%–79%, and <60% of total possible points (49). Importantly, although the search strategy and eligibility criteria did not address methodological design nearly all included studies were cross-sectional and the nature of cross-sectional studies limits conclusions on causality (50).

### 2.4 Data extraction and synthesis

From each included study, three researchers (A.H. and J.M.) independently extracted the following data: author names, title and year of publication, sample size, description of participants, survey instrument to assess grit and/or resilience, PA measures, statistical approaches, results, and key findings. A third reviewer (M.S.) then reviewed any inconsistencies and facilitated discussion to resolve discrepancies. All differences in data extraction were discussed and reconciled, with consensus achieved in all cases. Due to the overall heterogeneity of the methodology utilized in the included studies only a qualitative synthesis was performed. The synthesis of data was performed in several steps. First, the characteristics of included studies were aggregated by overall sample, country in which study was conducted, relevant socio-demographic factors (e.g., age, sex, education). Next, the specific grit and resilience instruments used in the included studies were summarized. In the last step, focused on the overall aims of the systematic review, the study findings related PA adherence, intensity, and domains were synthesized separately for grit and resilience.

## 3 Results

**Figure 2:**
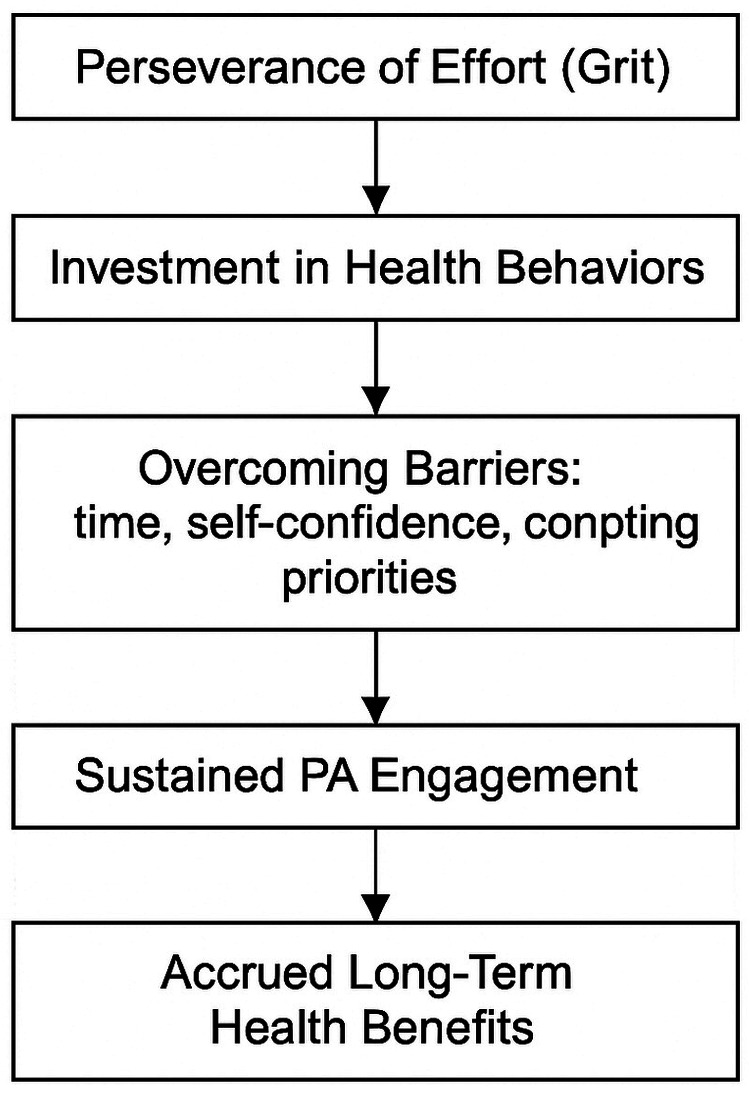
Search flow diagram.

The PRISMA search diagram in Figure 2 details the results of the search. Full text screening was conducted on 86 publications, and 60 publications were excluded for the following reasons: participants reported mental health (*n* = 2), chronic disease (*n* = 10), did not report a relationship between grit and PA (*n* = 45), and included participants younger than 18 (*n* = 3). Forward (*n* = 3) and backward (*n* = 4) citation searches then yielded additional studies leading to a total of 33 included studies in the systemic search. However, one published manuscript (15) reported results of 4 separate studies: (1) US adults only; (2) military, veterans, and civilians; (3) college students; (4) performing artists, while another study reported data on two studies (51). Since each study had unique results, in this manuscript the studies from Flinchbaugh et al. (51) and Martin et al. (15) are reported as separate studies [e.g., Martin et al. (15)-study 1, Martin et al. (15)-study 2, etc.]. Unless otherwise noted, all percentages reported in this study are reported as a percentage of 37 total studies [31 separate manuscripts + 2 studies extracted from Flinchbaugh et al. (51) and 4 studies extracted from Martin et al. (15)].

### 3.1 Methodological quality

The average quality score of the studies was 81.2 (±7.5)% with the minimum and maximum scores being 65% and 100%, respectively (Table 1). A majority (*n* = 28/37) studies were classified as being of high methodological quality. Items 13 and 14 were N/A and most of the articles scored low on items 3 and 7. Item 3 pertained to size justification reporting and ∼70% of the studies (*n* = 23/33) did not report a size justification or what methods were used to determine the sample size, thus resulting in a score of 0. Items 7, 13 and 14 on the AXIS tool are related to assessing and reporting non-responders and, in 60% of the included studies (*n* = 20/33), item 7 was not addressed and only 1 study addressed items 13 and 14 (Table 1). As previously mentioned, the inherent limitation of cross-sectional studies is inability to draw causality from the findings (50), which bears consideration when interpreting the methodological quality of the studies.

**Table 1:** Quality appraisal of included studies using appraisal tool for cross-sectional studies (AXIS).

Author(s)	Items																				Total Points	%
	Introduction	Methods										Results					Discussion		Other			
	1	2	3	4	5	6	7	8	9	10	11	12	13	14	15	16	17	18	19	20		
Studies using only a grit instrument (*n* = 11)																						
Allee et al. (52)	1	1	0	1	1	1	1	1	1	1	1	1	0	0	1	1	1	1	1	1	**15**	75%
Benedict et al. (53)	1	1	1	1	1	1	0	1	1	1	1	1	0	0	1	1	1	1	1	1	**17**	85%
Cazayoux and DeBeliso (54)	1	1	0	1	0	0	0	1	1	1	1	0	0	0	1	1	1	1	1	1	**13**	65%
Daniels et al. (55)	1	1	1	1	1	1	1	1	1	1	1	1	0	0	1	1	1	1	1	1	**18**	90%
Flinchbaugh et al. (51)	1	1	1	1	1	1	1	1	1	1	1	1	0	0	1	1	1	1	1	1	**18**	90%
Kelly et al. (56)	1	1	0	1	1	1	0	1	1	1	1	1	0	0	1	1	1	0	1	1	**15**	75%
Martin et al. (28)	1	1	1	1	0	1	0	1	1	1	1	1	0	0	1	1	1	1	1	1	**16**	80%
Martin et al. (15)	1	1	0	1	1	1	0	1	1	1	1	1	0	0	1	1	1	1	1	1	**16**	80%
Nothnagle and Knoester (38)	1	1	0	1	1	1	1	1	1	1	1	1	0	0	1	1	1	1	0	0	**15**	83%
Reed (57)	1	1	1	1	1	1	1	1	1	1	1	1	0	0	1	1	1	1	1	0	**17**	85%
Shamshirian et al. (58)	1	1	0	1	0	0	0	1	1	1	1	1	0	0	1	1	1	1	0	0	**12**	60%
Totosy de Zepetnek et al. (16)	1	1	0	1	1	1	0	1	1	1	1	1	0	0	1	1	1	1	1	1	**17**	85%
Studies using only a resilience instrument (*n* = 18)																						
Blanco-García et al. (59)	1	1	0	1	1	1	1	1	1	1	1	1	0	0	1	1	1	1	1	1	**17**	85%
Carriedo et al. (60)	1	1	0	1	1	1	0	1	1	1	1	1	0	0	1	1	1	1	1	1	**16**	80%
Chow and Cho (61)	1	1	1	1	1	1	1	1	1	1	1	1	0	0	1	1	1	1	1	1	**18**	90%
Harman et al. (62)	1	1	0	1	1	1	0	1	1	1	1	1	0	0	1	1	1	1	1	1	**16**	80%
Li et al. (63)	1	1	0	1	0	1	1	1	1	1	1	1	0	0	1	1	1	1	1	1	**17**	85%
Lines et al. (64)	1	1	0	1	0	0	0	1	1	1	1	1	0	0	1	1	1	1	0	1	**13**	65%
Martínez-Moreno et al. (46)	1	1	0	1	1	1	1	1	1	1	1	1	0	0	1	1	1	1	1	1	**15**	75%
Martínez-Moreno et al. (65)	1	1	0	1	1	1	0	1	1	1	1	1	0	0	1	1	1	1	1	1	**16**	80%
Ozkara et al. (66)	1	1	0	1	1	1	0	1	1	0	0	1	0	0	1	1	1	0	1	1	**13**	65%
Peyer et al. (67)	1	1	1	1	1	1	1	1	1	1	1	1	0	0	1	1	1	1	1	1	**18**	90%
Roebuck et al. (68)	1	1	1	1	1	1	0	1	1	1	1	1	0	0	1	1	1	1	1	1	**17**	85%
San Román-Mata et al. (69)	1	1	0	1	1	1	1	1	1	1	1	1	0	0	1	1	1	1	1	1	**15**	75%
Seçer and Çakmak Yıldızhan (70)	1	1	0	1	1	1	0	1	1	1	1	1	0	0	1	1	1	0	0	1	**14**	70%
Thogersen-Ntoumani et al. (71)	1	1	0	1	1	1	0	1	1	1	1	1	0	0	1	1	1	1	1	1	**16**	80%
To et al. (72)	1	1	0	1	1	1	0	1	1	1	1	1	0	0	1	1	1	1	1	1	**16**	80%
Wermelinger Ávila et al. (73)	1	1	0	1	0	1	1	1	1	1	1	1	0	0	1	1	1	1	1	1	**16**	80%
Xu et al. (74)	1	1	0	1	1	1	0	1	1	1	1	1	0	0	1	1	1	1	1	1	**17**	85%
Yu and Ye (75)	1	1	0	1	1	1	1	1	1	1	1	1	0	0	1	1	1	1	1	1	**17**	85%
Studies using a grit and resilience instrument (*n* = 3)																						
Atkinson and Martin (76)	1	1	1	1	1	1	1	1	1	1	1	1	1	1	1	1	1	1	1	1	**19**	95%
Dunston et al. (14)	1	1	1	1	1	1	0	1	1	1	1	1	0	0	1	1	1	1	1	1	**17**	85%
Schaefer et al. (77)	1	1	0	1	1	1	0	1	1	1	1	1	0	0	1	1	1	1	1	1	**16**	80%

Note: Bold values indicate the total score on the 20 point scale of the appraisal tool for cross-sectional studies (AXIS).

### 3.2 Study characteristics

The characteristics of the included study reports are summarized in Table 2. An important characteristic of the body of literature is related to the year of publication. Despite the search range including the past 30 years, the earliest study was published in 2014 (57) with ∼88% of the studies being published in the last 5 years (Table 2).

**Table 2:** Characteristics of included studies.

Authors, Year	Continent/Country	Participants	Study Design	Grit/Resilience Instrument	PA Measure(s)	Statistical Analysis	Results	Key Findings
(A) Studies using only a grit instrument (*n* = 11)								
Allee et al. (52)	North America/United States	First-semester university students *n*: 431 Sex: m = 169, f = 262 Age: 19.3 ± 2.4 years	Cohort-type observational study, cross-sectional	12-item Grit Scale	Daily Step Count via wearable (Fitbit)	Bivariate correlation Multiple regression *DV* *=* *Grit* *IV’s* *=* *Step count, Age, Sex*	Grit was not associated with daily step count in the bivariate analysis (*r* = 0.11, *p* = 0.062). After controlling for age and sex, the relationships between grit and daily step count were significant (*β* = 509.28, *p* = 0.0496).	In university students, only after controlling for sex and age, steps per day was significantly associated with grit.
Benedict et al. (53)	North America/United States	US Army ranger school candidates *n*: 670 Sex: m = 614, f = 19 Age: 25.3 ± 4.2 years	Cross-Sectional	8-item Grit Scale	Self-reported months of strength training, minutes per week of strength training, and days per week of rucking to train for ranger school.	Bivariate correlation	Grit was associated with minutes per week of strength training (*r* = 0.09, *p* < 0.05), and days per week ruck marching (*r* = 0.08, *p* < 0.05) but not months of strength training (*r* = 0.07, *p* > 0.05).	In US Army ranger school candidates, greater levels of grit were found to have a weak, positive relationship with some, but not all physical training leading up to ranger school.
Cazayoux and DeBeliso (54)	North America/United States	CrossFit athletes, n:50; Advanced*, *n* = 23, Novice, *n* = 27. Sex: not reported Age: not reported *Advanced was defined as competing at the CrossFit games. Novice performed CrossFit workouts recreationally at a local gym.	Cross-sectional	12-item Grit Scale	Performance achievement in CrossFit	t-tests *DV* *=* *Grit, persistence of effort, consistency of interest* *IV* *=* *Dichotomous variable for CrossFit performance level (Novice vs. Advanced)*	Advanced CrossFit athletes were found to have great total grit (*p* = 0.03, *d* = 0.57) and consistency of interest subscale (*p* = 0.02, *d* = 0.59) scores; however, no difference in persistence of effort was found (*p* = 0.13, *d* = 0.38)	The findings support the importance of grit in athletic achievement within the sport of CrossFit.
Daniels et al. (55)	North America/United States	Undergraduate university students *n*: 875 Sex: m = 500, f = 375, Age: 20.0 ± 2.5 years	Cross-Sectional	8-item Grit Scale	International Physical Activity Questionnaire (31-item)	Multiple regression DVs: *Grit, perseverance of effort, consistency of interest* IVs: Total MET-min/week; Vigorous MET min/week; Moderate MET-min/week; Walking MET-min/week; Work MET-min/week; Active transport MET-min/week; Domestic MET-min/week; Leisure MET-min/week *For each of the 3 DV and 8 IV, multiple regression models were created that controlled for sex and age.	Grit was positively and significantly associated with total *MET-min/*week (*β* = 1,009.99, *p* < 0.001), *Vigorous MET min/week* (*β* = 470.09, < 0.001), *Moderate MET-min/week* (*β* = 306.94, *p* = 0.001), walking *MET-min/week* (*β* = 232.96, *p* = 0.018). domestic *MET-min/week* (*β* = 192.14, *p* = 0.007) *and leisure MET-min/week* (*β* = 555.42, *p* < 0.001). Consistency of interest was positively and significantly associated with total *MET-min/*week (*β* = 307.81, *p* = 0.047) and Vigorous *MET-min/week* (*β* = 169.40, *p* = 0.019). Perseverance of effort was significantly associated with total *MET-min/week* (*β* = 756.95, *p* < 0.001), Vigorous *MET-min/week* (*β* = 318.87, *p* < 0.001), Moderate *MET-min/week* (*β* = 194.91, *p* = 0.025), walking *MET-min/week* (*β* = 243.17, *p* = 0.006), active transport *MET-min/week* (*β* = 132.44, *p* = 0.033), domestic *MET-min/week* (*β* = 160.92, *p* = 0.012), and leisure *MET-min/week* (*β* = 428.32, *p* < 0.001).	In US university students, grit and perseverance positively correlated with various PA intensities and domains. Specifically, consistency of interest showed a positive association with total PA and VPA, while the perseverance of effort component exhibited a stronger influence on PA outcomes than consistency of interest.
Flinchbaugh et al. (51) Study 1	North America/United States	Working Adults *n* = 207 Sex: m = 91, f = 116 Age: 44.7 ± 13.2 years	Cross-Sectional	12-Item Grit Scale	Weekly volume of PA	Structural Equation Modeling (SEM) *Weekly physical activity volume (observed) predicting consistency of interest (latent) and perseverance of effort (latent)*	Physical activity was negatively associated with consistency of interest (*β* = −0.14, *p* < 0.05) and not associated with perseverance of effort. The SEM demonstrated good fit indices [*χ*^2^(46) = 89.77, *p* = 0.00; RMSEA = 0.07, 90% CI = [.05 to.09]; CFI = 0.97; TLI = 0.96]	In physically active working adults, the subscales of grit were not found to have a positive relationship with weekly PA.
Flinchbaugh et al. (51) Study 2	North America/United States	Competitive cyclists *n* = 119 Sex: m = 61, f = 58 Age: 43.2 ± 10.8 years	Cross-Sectional	12-Item Grit Scale	Weekly cycling training volume	Structural Equation Modeling (SEM) *Weekly training volume (observed) predicting consistency of interest (latent) and perseverance of effort (latent)*	Weekly training volume was positively associated with consistency of interest (*β* = 0.27, *p* < 0.05) and with perseverance of effort (*β* = 0.25, *p* < 0.05). The SEM demonstrated good fit indices [*χ*^2^(94) = 148.52, *p* = 0.00; RMSEA = 0.08, 90% CI = [.05 to.10]; CFI = 0.91; TLI = 0.88]	In competitive cyclists, who were also working, the subscales of grit were found to have a positive relationship with weekly cycling training volume.
Kelly et al. (56)	North America/United States	United States Military Academy Cadets Total *n* = 2,558 m = 2,188, f = 370 Class 1 n: 1,310 entered and 1,046 graduated Sex: 14% female Age: 19 years (Mean)	Cross-Sectional	12-Item Grit Scale	Physical Program Score (PPS)	Bivariate correlation	PPS was positively associated with total grit (*r* = 0.09, *p* < 0.01), consistency of interest (*r* = 0.06, *p* < 0.01), and consistency of effort (*r* = 0.10, *p* < 0.01).	Grit total score and subscales were found to have a positive relationship with a composite measure of physical performance (academic, fitness testing and competitive sports activity) in West Point cadets.
Martin et al. (28)	North America/United States	University Students *n* = 125 Sex: m = 61, f = 64 Age: 19.4 ± 0.9 years (all between 18 and 22 years)	Cross-sectional	8-item Grit Scale	International Physical Activity Questionnaire Short Form	Stepwise multiple linear regression *DV: Grit* *Model 1 IVs: VPA, MPA, LPA* *Model 2 IVs: model 1* *+* *sitting time* *Model 3 IVs: model 2* *+* *sleep quality* *Model 4 IVs: model 3* *+* dietary behaviors. *Gender, employment, and population density were controlled for in all models.	Grit was positively associated with VPA in model 1 (*β* = 0.254, *p* < 0.05) and 2 (*β* = 0.245, *p* < 0.05). None of the other PA variables were significantly associated with grit in any of the models.	In US university students during the COVID-19 pandemic, grit and VPA were found to have a positive relationship; however, when sleep and dietary behaviors were accounted for the relationship was no longer significant. The final regression model indicated that grit was positively associated with better sleep quality and dietary behaviors but not with any measure of PA.
Martin et al. (15) Study 1	North America/United States	Adults *n* = 1,388 Sex: m = 393, f = 995 Age: 33.9 ± 13.9 years	Cross-sectional	8-item Grit Scale	International Physical Activity Questionnaire Short Form	Multiple regression *DV* *=* *Grit* *IV’s* *=* MVPA, LPA, Sitting time, dietary behaviors *Age and gender were controlled for	Grit was positively associated with MVPA (*β* = 0.093, *p* < 0.05).	In several samples of US adults during the COVID-19 pandemic, grit was associated with MVPA in populations who did not currently, or previously, have occupational requirements to be physically active.
Martin et al. (15) Study 2	North America/United States	Active-duty military *n* = 253 Sex: m = 166, f = 87 Age: 33.4 ± 7.3 years Veterans *n* = 169 Sex: m = 110, f = 59 Age: 42.6 ± 9.9 years Civilians *n* = 388 Sex: m = 104, f = 284 Age: 39.2 ± 12.1 years	Cross-sectional	8-item Grit Scale	International Physical Activity Questionnaire Short Form	Multiple regression *DV* *=* *Grit* *IV’s* *=* MVPA, LPA, Sitting time, dietary behaviors *Age and gender were controlled for. Separate linear regression models were created for active-duty military, veterans, and civilians.	Grit was positively and significantly associated with MVPA in civilians (*β* = 0.252, *p* < 0.001) but not associated in active-duty members of the military (*β* = −0.097, *p* > 0.05) or veterans *β* = 0.147, *p* > 0.05).	
Martin et al. (15) Study 3	North America/United States	University Students *n* = 144 Sex: m = 66, f = 78 Age: 19.5 ± 0.9 years	Cross-sectional	8-item Grit Scale	International Physical Activity Questionnaire Short Form	Multiple regression *DV* *=* *Grit* *IV’s* *=* MVPA, LPA, Sitting time, dietary behaviors *Age and gender were controlled for	Grit was positively associated with MVPA (*β* = 0.185, *p* < 0.05).	
Martin et al. (15) Study 4	North America/United States	Performing Artists *n* = 77 Sex: m = 16, f = 61 Age: 36.1 ± 12.7 years	Cross-sectional	8-item Grit Scale	International Physical Activity Questionnaire Short Form	Multiple regression *DV* *=* *Grit* *IV’s* *=* MVPA, LPA, Sitting time, dietary behaviors *Age and gender were controlled for	Grit was not associated with MVPA (*β* = −0.21, *p* > 0.05).	
Nothnagle and Knoester (38)	North America/United States	Adults *n* = 3,931 Sex: m = 1,022, f = 2,909 Age: 21–65 years	Cross-sectional	8-item Grit Scale	2018–2019 National Sports and Society Survey on sports participation during childhood and adulthood survey	Multiple regression *DV* *=* *Grit* *IV’s* *=* Participation in sport as a child, Continual childhood sport participation, Regular participation in sport as an adult during the past year. *Age and gender were controlled for	Grit was positively associated with childhood sport participation (*β* = 0.06, *p* < 0.05), continual childhood sport participation (*β* = 0.21, *p* < 0.001), and regular participation in sport during the past year as an adult (*β* = 0.11, *p* < 0.001).	Greater levels of grit were observed in adults who regularly participate in sport and those who continually participated in sport as a child.
Reed (57)	North America/United States	University students, faculty, and staff *n* = 1,171 Sex: m = 406, f = 755 Age: 34.6 ± 14.0 years	Cross-Sectional	8-item Grit Scale	Self-reported exercise behavior scores calculated as: Exercise Behavior = (months × LPA) + (months × MPA) + (months × VPA). *Months were how many months participants had been exercising at each intensity 1 or more times per week for more than 15 min.*	Bivariate correlation Multiple regression *DV* *=* *Exercise behavior* *IV’s* *=* Grit *Age and gender were controlled for t-tests *DV: Grit* *IV: Exercise behavior as dichotomous variable (exercisers vs. non-exercisers)*	Bivariate correlation analysis found that grit was positively associated with exercise score (*r* = 0.18, *p* < 0.001). Multiple regression analysis found that exercise behavior was positively associated with grit (*β* = 14.50, *p* = 0.035). Participants who engaged in exercise (=989) had greater grit (*p* < 0.001, *d* = 0.34) than those who do not engage in exercise (*n* = 172).	In healthy adults, attending school and working at a university, several statistical approaches all indicated that greater levels of grit were found in those individuals who engaged in more PA.
Shamshirian et al. (58)	Asia/Iran	Elite level wrestlers *n* = 117 Sex: m = 117, f = 0 Age: 22.0 ± 4.5 years Non-wrestler controls *n* = 102 Sex: m = 56, f = 46 Age: 25.5 ± 3.9 years	Cross-sectional	8-item Grit Scale	Participation in elite level athletics (wrestling) vs. control group of similar age and culture university students.	t-test *DV: Grit* *IV: Wrestler vs. University student*	The mean (SD) of grit scores were 4.09 (0.59) and 3.45 (0.63) for wrestlers and university students, respectively. Wrestlers had significantly greater grit (*p* < 0.001) than university students.	High level Iranian wrestlers more passion and grit than the control group of Iranian university students.
Totosy de Zepetnek et al. (16)	North America/United States	Adults *n* = 888 m: 237, f = 651 Age: 34.8 ± 14.0 years	Cross-sectional	8-item Grit Scale	International Physical Activity Questionnaire Short Form	Multiple regression *DV* *=* *Grit* *IV’s* *=* VPA, MPA, LPA *Age and gender were controlled for *ANCOVA* *DV* *=* *Grit* *IV: PA (4 levels: 0* *min/week,* *<* *150* *min/week, 150 to <300* *min/week, 300* *+* *min/week), Covariates: sex, education, employment/student status and any chronic disease*	The regression model indicated grit was positively associated with VPA (*β* = 0.077, *p* < 0.001 but not MPA (*p* > 0.05) or LPA (*p* > 0.05). Participants reporting 300 + minutes/week of PA had significantly greater grit than participants reporting 0 min/week, < 150 min/week or 150 to <300 min/week of PA.	In U.S. adults during the COVID-19 pandemic the study found a positive correlation between Grit and high-intensity physical activity (e.g., VPA). Additionally, individuals who participated in high volumes of physical activity (300 + min/week) exhibited significantly greater levels of grit compared to those classified in other categories of weekly physical activity volume, ranging from inactive to meeting the 150 min/week guidelines.
(B) Studies using only a resilience instrument (*n* = 18)								
Blanco-García et al. (59)	Europe/Spain	Competitive athletes *n* = 1,047 Sex: m = 589, f = 458 Age: m, 24.4 ± 6.0 years; f, 24.1 ± 5.8 years Sports represented: Basketball *n* = 165 Handball *n* = 165 Volleyball *n* = 158 Athletics *n* = 242 Judo *n* = 317	Cross-sectional	Brief Resilience Scale	Participation in sport	*ANCOVA 1* *DV* *=* *Resilience* *IV: Sport (5 levels: basketball, athletics, handball, volleyball, judo)* *ANCOVA 2* *DV* *=* *Resilience* *IV: sport category (3 levels: team, individual, and combat)* *ANCOVA 3* *DV* *=* *Resilience* IV: Sport type (2 levels: team and non-team) *Age was the covariate in all analyses	There were no significant differences in resilience between individuals based on sport played or sport category.	In young Spanish adults participating in competitive sport, the study found that the specific sport or type of sport did not exert any discernible impact on resilience levels among participants.
Carriedo et al. (60)	Europe/Spain	Adults *n* = 1,795 Sex: m = 656, f = 1,150 Age: 40.5 ± 15.7 years	Cross-Sectional	Connor-Davidson Resilience Scale *Focused on locus of control, self-efficacy, and optimism sub-components of resilience instead of overall resilience.	International Physical Activity Questionnaire Short Form	Bivariate correlation Multiple regression Model 1 *DV* *=* *Locus of control* *IVs* *=* *VPA, MPA, LPA* Model 2 *DV* *=* *Self-efficacy* *IVs* *=* *VPA, MPA, LPA* Model 3 *DV* *=* *Optimism* *IVs* *=* VPA, MPA, LPA *Controlled for age and gender in models	Bivariate correlations indicated that 1) VPA was positively correlated with locus of control (*r* = 0.09, *p* < 0.01), self-efficacy (*r* = 0.09, *p* < 0.01) and optimism (*r* = 0.08, *p* < 0.01); MPA was positively correlated with locus of control (*r* = 0.08, *p* < 0.01), self-efficacy (*r* = 0.07, *p* < 0.01) and optimism (*r* = 0.06, *p* < 0.01); and LPA was positively associated with locus of control (*r* = 0.05, *p* < 0.05). The regression models indicated that (1) locus of control was positively associated with VPA (*β* = 0.040, *p* < 0.01) and LPA (*β* = 0.031, *p* < 0.05); (2) self-efficacy was positively associated with VPA (*β* = 0.028, *p* < 0.05) and (3) optimism was negatively associated with VPA (*β* = −0.008, *p* < 0.05) and MPA (*β* = −0.344, *p* < 0.001) but positively associated with LPA (*β* = 0.004, *p* < 0.05).	In Spanish adults during the COVID-19 pandemic, VPA was the only PA intensity level associated with all 3 components of resilience examined in the multiple regression models. There was a positive relationship between locus of control and self-efficacy components of resilience with VPA; however, when demographic factors were accounted for there was a negative relationship between the optimism component of resilience and VPA.
Chow and Choi (61)	Asia/China	University students *n* = 416 Sex: m = 167, f = 249 Age: Not reported	Cross-Sectional	Brief Resilience Scale	Godin-Shephard Leisure Time Physical Activity Questionnaire	Bivariate correlation	The correlation between PA and resilience was not significant.	In Chinese university students attending school in Hong Kong the study did not find a significant relationship between resilience and PA.
Harman et al. (62)	Europe/Primarily France (73%) and United Kingdom (11%)	Endurance sport athletes *n* = 3,551 Sex: m = 2,975, f = 576 Age: 44.1 ± 9.8 years *Note: Participants self-reported their athletic level as amateur, competitive amateur, or semi-professional*/*professional athlete.*	Cross-sectional	Connor-Davidson Resilience Scale	Self-Reported Weekly PA and Competitive Level	Bivariate correlation (Spearman’s) Multiple regression *DV* *=* *Perceived barriers to training during the COVID-19 pandemic lockdown* *IV* *=* *training volume*	Athletic level was positively associated with resilience (*r*s = 0.11, *p* < 0.001). Perceived barriers to training were associated with resilience (*β* = 0.003, *p* < 0.05) such that greater resilience led to fewer perceived barriers.	During the COVID-19 lockdown European endurance athletes with greater resilience perceived fewer barriers to training. Resilience had a positive relationship with achieving greater success (e.g., amateur, competitive amateur, professional) in endurance sport within the sample.
Li et al. (63)	Asia/China	University students *n* = 1,214 Sex: m = 506, f = 708 Age: 20.0 ± 1.7 years	Cross-sectional	Connor-Davidson Resilience Scale	Physical Exercise Questionnaire with dimensions for exercise adherence and commitment	Bivariate correlation Structural Equation Modeling (SEM) *Physical exercise (observed) predicting resilience (observed)*	Physical exercise was positively associated with resilience (*r* = 0.47, *p* < 0.001). Resilience was positively associated with resilience (*β* = 0.51, *p* < 0.01). The SEM demonstrated good fit indices [*χ*^2^(46) = 4.698; CFI = 0.993; GFI = 0.988; AGFI = 0.970, NFI = 0.991; IFI = 0.993; RMSEA = 0.055].	In Chinese university students physical exercise was positively related to resilience during the COVID-19 lockdown.
Lines et al. (64)	Oceania/Australia	University students *n* = 52 Sex: m = 11, f = 41 Age: 21.9 ± 4.6 years	Longitudinal Design with 3 measurement periods over 6 days. Each measurement period was separated by 8. Weeks. Time point 1 occurred in the middle of a semester, time point 2 during the study week prior to finals and time point 3 during the first week of the next semester.	Brief Resilience Scale	Actigraphy via 24-hour Triaxial Accelerometry to record LPA, MPA and VPA.	Bivariate correlations at each time point.	There was a positive association between VPA and resilience at time point 1 (*r* = 0.31, *p* < 0.01) but non-significant associations at time points 2 and 3. There was a positive association between MPA and resilience at time point 1 (*r* = 0.33, *p* < 0.01) and time point 2 (*r* = 0.13, *p* < 0.05) but a non-significant association at time point 3. There was a positive association between LPA and resilience at time point 1 (*r* = 0.26, *p* < 0.01) and time point 2 (*r* = 0.18, *p* < 0.01) but a non-significant association at time point 3.	In a cohort of Australian university students relationships between VPA, MPA and LPA with resilience differed at various points in the semester.
Martínez-Moreno et al. (46)	Europe/Spain	Older Adults *n* = 381 Sex: m = 210, f = 171 Age: 68.1 ± 8.6 years	Cross-sectional	Connor-Davidson Resilience Scale	Participants self-reported PA behaviors regarding engaging in PA (yes or no) and for those who were physically active how many days per week they engaged in PA.	t-test *DV: Resilience* *IV: Engaging or not in PA*	Resilience scores were higher in those who engaged in PA (*p* < 0.001).	Older Spanish adults who engaged in PA had higher scores in resilience than those who did not.
Martínez-Moreno et al. (65)	Europe/Spain	Older Adults, *n* = 381 Sex: m = 191, f = 190 Age: 68.8 ± 8.7 years	Cross-sectional	Connor-Davidson Resilience Scale	Participants self-reported PA behaviors regarding engaging in PA (yes or no) and for those who were physically active how many days per week they engaged in PA.	Bivariate Correlation t-test *DV: Resilience* *IV: Engaging or not in PA*	Resilience scores were higher in those who engaged in PA (*p* = 0.016). There was no association between resilience and the number of days per week they engaged in PA.	Older Spanish adults who engaged in PA had higher scores in resilience than those who did not. However, there was no significant relationship between resilience and the number of days per week PA was performed. .
Ozkara et al. (66)	Europe/Turkey	University Students *n* = 331 Sex: m = 197, f = 134 Age: Not reported	Cross-Sectional	Brief Resilience Scale	Childhood and Adolescence Physical Activity Levels Questionnaire and Childhood	Bivariate Correlation Univariate Regression *DV: Resilience* *IV: PA*	PA was positively associated with resilience based on bivariate correlation (*r* = 0.598, *p* < 0.001) and univariate regression (*β* = 0.598, *p* = 0.016) analyses.	In Turkish university students, who were preservice physical education teachers, resilience was positively associated with PA.
Peyer et al. (67)	North America/United States	University Students *n* = 300 Sex: m = 74, f = 226 Age: Not reported	Cross-Sectional	Brief Resilience Scale	International Physical Activity Questionnaire	Bivariate Correlation 2-way ANOVA *DV: Resilience* *IV1: Meeting PA Guidelines (2 levels: Yes, No)* *IV2: Gender (2 levels: Yes, No)*	Resilience was positively correlated with days per week of strength training (*r* = 0.18, *p* < 0.01) and minutes per week of strength training (*r* = 0.15, *p* = 0.03) in females. Resilience was positively correlated with days per week of moderate exercise (*r* = 0.40, *p* < 0.01), days per week of strength training (*r* = 0.26, *p* = 0.03), minutes per week of moderate exercise (*r* = 0.33, *p* < 0.01), and minutes per week of walking (*r* = 0.27, *p* = 0.02) in males. There was a significant main effect of Gender (male > female resilience) and interaction of Meeting PA Guidelines × Gender in the 2-way ANOVA model with males meeting PA guidelines reporting the greatest resilience and females not meeting PA guidelines reporting the least resilience.	In United States university students during the COVID-19 pandemic, resiliency was related to frequency and minutes per week of strength training in females. Resiliency was also related to moderate exercise frequency and minutes per week, frequency of strength training per week, and minutes of walking per week in males. Males meeting PA guidelines reporting the greatest resilience and females not meeting PA guidelines reporting the least resilience.
Roebuck et al. (68)	Oceania/Australia	Ultra-runners *n* = 20 Sex: m = 9, f = 11 Age: 42.6 ± 7.9 years Controls *n* = 20 Sex: m = 9, f = 11 Age: 40.2 ± 8.5 years	Cross-Sectional	Connor-Davidson Resilience Scale	Self-reported exercise behaviors	t-tests *DVs: Resilience, time spent running per week, total distance run per week, weekly strength training frequency, weekly stretching frequency* *IV: Ultra-runners vs. non-running controls.*	Ultra-runners had significantly higher resilience scores than the control group (*p* = 0.014, *d* = 0.81). In a week, ultra-runners reported more time spent running (*p* < 0.001), total distance run (*p* < 0.001), frequencies of strength (*p* = 0.002), and stretch training (*p* = 0.046) than non-running controls.	Compared to controls, ultra-runners had significantly higher resilience, spent significantly more time running, covered more distance running, and had higher frequency of strength training and stretching per week.
San Román-Mata et al. (69)	Europe/Spain	University Students *n* = 1,095 Sex: m = 351, f = 744 Age: 21.4 ± 4.6 years	Cross-sectional	Connor-Davidson Resilience Scale	Ad-Hoc Questionnaire based on WHO physical activity recommendations.	t-tests *DVs: Resilience and subcomponents* *IV: Meeting PA Guidelines (2 levels: Yes, No).*	Those who meet the minimum PA guidelines have greater overall resilience (*p* < 0.01 m *d* = 0.22) and greater scores in the resilience subcomponents of locus of control and commitment (*p* < 0.01, *d* = 0.25), self-efficacy and resistance to discomfort (*p* < 0.01, *d* = 0.24), and optimism and adaption to stressful situations (*p* < 0.01, *d* = 0.28).	University students in Spain who met the minimum recommended level of PA per week reported greater total resilience compared to those who did not meet the PA recommendations.
Seçer and Çakmak Yıldızhan (70)	Europe & Asia/Turkey	University students *n* = 1,734 Sex: m = 725, f = 1,009 Age: Mean not reported; 16.7% were 17–19 years; 46.7% were 20–21 years and 36.6% were 22 + years	Cross-sectional	Psychological Resilience Scale	International Physical Activity Questionnaire Short Form	Bivariate Correlation Univariate Regression *DV: Resilience* *IV: PA (MET-min/week)*	Bivariate correlations indicated a positive correlation between PA and total resilience (*r* = 0.17, *p* < 0.01) and sub-dimensions of resilience of self-commitment (*r* = 0.14, *p* < 0.01), challenge (*r* = 0.13, *p* < 0.01) and locus of control (*r* = 0.12, *p* < 0.01). PA was a significant predictor of resilience (*β* = 0.176, *p* < 0.01).	In a sample of university students attending school in Turkey, there was a positive relationship between PA, total resilience, and resilience subcomponents. PA was a significant predictor of resilience.
Thogersen-Ntoumani et al. (71)	Europe/United Kingdom	Older manual workers *n* = 217 Sex: m = 149, f = 68 Age: 57.1 ± 5.6; range, 50–77 years	Cross-sectional	The Brief Resilience Scale	Baecke Questionnaire	Bivariate Correlation	The correlations between resilience with work, leisure, and sport/exercise PA were not statistically significant (*p* > 0.05).	In older manual workers in the UK there was no association between resilience with work, leisure, or sports/exercise domains of PA.
To et al. (72)	Oceania/Australia	Adults Timepoint 1 Survey *n* = 638 Sex: m = 199, f = 436 Age: 52.5 ± 14.3 years Timepoint 2 Survey *n* = 843 Sex: m = 269, f = 573 Age: 53.2 ± 14.1 years Timepoint 3 Survey *n* = 545 Sex; m = 161, f = 382 Age: 53.8 ± 13.9 years	Longitudinal and Cross-sectional across 3 time points. Time point 1 occurred in April 2020, time point 2 occurred in August 2020 and time point 3 occurred in December 2020.	Brief Resilience Scale	Active Australia Survey (LPA, MPA, VPA over past 7 days)	Longitudinal data: Linear mixed models Cross-sectional data: Linear models *DV: Resilience* IV: Meeting PA Guidelines (2 levels: Yes, No). *Controlled for age, gender, years of education, household income, marital status, chronic disease, depression, anxiety, and stress levels in models.	Meeting MVPA guidelines resulted in higher resilience in the longitudinal (aDif = 0.07, *p* < 0.05, 95% CI = 0.01, 0.13) and cross-sectional samples (aDif = 0.15, *p* < 0.001, 95% CI = 0.08, 0.21). *Note: aDif stands for adjusted differences between meeting vs. not meeting MVPA guidelines in models adjusted for covariates.*	In Australian adults during the COVID-19 pandemic, the findings of the study indicate that participants that obtained at least 150 MVPA minutes per week had higher resilience scores.
Wermelinger Ávila et al. (73)	South America/Brazil	Older adults *n* = 180 Sex: m = 25, f = 155 Age: 69.6 ± 6.3 years	Longitudinal across 4 years with 3 time points. Time point 1 was 2015, time point 2 and 3 were 2- and 4-year follow ups, respectively.	Wagnild and Young’s Psychological Resilience Scale	International Physical Activity Questionnaire *Participants were classified as “regularly” active (e.g., meeting MVPA guidelines for all 3 time points) and ‘intermittently’ active (e.g., those not meeting MVPA guidelines in at least 1 time point.*	2 × 3 Repeated Measures ANCOVA *DV: Resilience* *IV1: PA (2 levels: Regularly Active, Intermittently Active)* IV2: Time (3 timepoints: baseline, 2-year follow-up, 4-year follow-up) *Age was included as a covariate.	There was a significant main effect for PA (*F* = 5.143, *p* = .025, *η*^2^ = 0.029) with higher levels of resilience in participants who were regularly active. There was no main effect of time (*F* = 0.222, *p* = 0.801, *η*^2^ = 0.001), or interaction effect of PA × Time (*F* = 0.319, *p* = 0.727, *η*^2^ = 0.002).	Older Brazilian adults who maintained regular PA were more resilient than those who did not maintain regular PA across a 4-year time period.
Xu et al. (74)	Asia/China	University students *n* = 2,375 Sex: m = 1,110, f = 1,265 Age: 20.3 ± 2.0 years	Cross-sectional	Connor-Davidson Resilience Scale	International Physical Activity Questionnaire Short Form	Bivariate Correlation Multiple regression *DV* *=* *Resilience* *IV* *=* PA *Controlled for age, gender, grade, major, residence and whether an only child in model Mediation analysis (Haye’s PROCESS macro) *PA (observed) predicting resilience (observed)*	PA was positively correlated with resilience (*r* = 0.159, *p* < 0.01). The regression analysis indicated a positive association of resilience with PA (*β* = 0.151, *p* < 0.001). PA had a direct mediating effect on resilience (*β* = 0.051, 95% CI = 0.021–0.081, *p* < 0.001).	In Chinese university students PA was found to have a positive relationship with resilience.
Yu and Ye (75)	Asia/China	University students *n* = 352 Sex: m = 131, f = 221 Age: 20.8 ± 2.2 years	Cross-Sectional	Connor-Davidson Resilience Scale	International Physical Activity Questionnaire—Short Form *Minimum and adequate levels of PA where definitions are provided in notes below.	Logistic regression *DVs* *=* *Minimum MPA, Minimum VPA, Minimum MVPA, Adequate MPA, Adequate VPA, Adequate MVPA* *IV* *=* Resilience *Controlled for age, gender, and BMI in model. *For each DV a separate model was computed.	Resilience was a significant predictor of obtaining minimum MPA (*β* = −0.041, OR = 0.960, *p* < 0.05), minimum MVPA (*β* = 0.024, OR = 1.024, *p* < 0.05), and adequate MVPA (*β* = 0.023, OR = 1.023, *p* < 0.05).	In Chinese university students, greater levels of resilience were associated with attaining minimum MVPA and adequate MVPA guidelines but decreased the odds of attaining minimum MPA guidelines. Notably, only 43.5% of the sample reported meeting minimum MVPA guidelines.
(C) Studies using a grit and resilience instrument (*n* = 3)								
Atkinson and Martin (76)	North America/United States	Wheelchair rugby athletes *n* = 87 Sex: m = 80, f = 7 Age: 35.9 ± 9.3 years	Cross-sectional	8-item Grit Scale Connor-Davidson Resilience Scale	16-item Athlete Engagement Questionnaire	Bivariate Correlation Multiple regression *DV* *=* *Sport engagement* *IVs* *=* *Grit, Resilience*	Grit was positively correlated with sport engagement (*r* = 0.33, *p* < 0.05) and resilience (*r* = 0.46, *p* < 0.05). Sport engagement was positively associated with grit (*β* = 0.21, *p* < 0.05) and resilience (*β* = 0.23, *p* < 0.05).	In wheelchair rugby athletes, greater grit and resilience had a positive relationship with sport engagement.
Dunston et al. (14)	North America/United States	University students *n* = 244 Sex: m = 79, f = 165 Age: 21.1 ± 2.9 years	Cross-sectional	8-item Grit Scale Connor-Davidson Resilience Scale	International Physical Activity Questionnaire Short Form	Bivariate Correlation Multiple regression and mediation analyses *DVs* *=* *Resilience, Consistency of interest, Perseverance of effort* *IVs* *=* VPA *Controlled for sex, year in school and GPA in models. 1-way ANCOVAs DVs = *Resilience, Consistency of interest, Perseverance of effort* IVs: Tertiles of MPA and VPA *Controlled for sex, year in school and GPA in models. **Tertiles of VPA and MPA were 1) 0 to *<* *75* *min/week, 2) 75* *+* *to <300* *min/week, and 3) 300* *+* *min/week.*	VPA was positively correlated with resilience (*r* = 0.16, *p* = 0.01), perseverance of effort (*r* = 0.20, *p* = 0.002), but negatively correlated with consistency of interest (*r* = −0.22, *p* = 0.002). MPA was positively correlated with perseverance of effort (*r* = 0.17, *p* = 0.007) and negatively correlated with consistency of interest (*r* = −0.15, *p* = 0.02). MPA and resilience were not found to be correlated nor was time spent walking or sitting. Regression analyses found that VPA was positively associated with resilience (*β* = 0.17, *p* = 0.01) and perseverance of effort (*β* = 0.18, *p* = 0.004) but negatively associated with consistency of interest (*β* = −0.22, *p* = 0.001), independent of demographic variables. 1-way ANCOVAs found differences in consistency of interest (*p* = 0.04, tertile 3 > tertile 1), perseverance of effort (*p* = 0.02, tertile 3 > tertile 1), and resilience (*p* = 0.007, tertile 2 > tertile 1) across tertiles. There were no differences across tertiles for MPA.	In US university students, the overall findings indicated that VPA showed positive associations with resilience and perseverance of effort but exhibited a negative association with consistency of interest. Higher levels of VPA were correlated with increased scores in grit and resilience, while MPA did not demonstrate similar significant relationships.
Schaefer et al. (77)	North America/United States	United States Military Academy Cadets *n* = 4,299 Sex: m = 3,396, f = 903 Age: Did not report	Cross-Sectional	8-item Grit Scale Dispositional Resilience Scale	Scores from Military Movement Course (MMC) aggregate and individual events (tumbling, high bar, trampoline, obstacle, strength, vault, rope climb).	Multivariate multiple regression DVs: Aggregate and Individual MMC event scores IVs; Grit, Resilience, Optimism *Controlled for race, gender, NCAA athlete status, interactions, graduation year, and GPA.	Grit was a significant predictor of aggregate MMC score (*p* < 0.001, *η*^2^ = 0.001) and the strength event (*p* = 0.016, *η*^2^ = 0.003). Resilience was a significant predictor of aggregate MMC score (*p* = 0.002, *η*^2^ = 0.006), tumbling (*p* = 0.045, *η*^2^ = 0.002), high bar (*p* < 0.001, *η*^2^ = 0.003), obstacle (*p* < 0.001, *η*^2^ = 0.004), strength (*p* = 0.016, *η*^2^ = 0.002), vault (*p* = 0.016, *η*^2^ = 0.002), and rope climb (*p* < 0.001, *η*^2^ = 0.004).	In US military academy cadets, grit and resilience significantly predicted aggregate MMC event score. However, in terms of individual MMC events, grit was only significantly related to a strength event whereas resilience was significantly related to all events of the MMC except the trampoline.

AGFI, adjusted goodness-of-fit index; ANCOVA, analysis of covariance; APFT, Army Physical Fitness Test; BMI, body mass index; CFI, comparative fit index; CI, confidence interval; DV, dependent variable; ES, effect size; f, female; GFI, goodness-of-fit index; GPA, grade point average IFI, incremental fit index; IV, independent variable; LPA, light physical activity; m, male; MET, metabolic equivalent; MMC, military movement course; MPA, moderate physical activity; MVPA, moderate to vigorous physical activity; NFI, normal fit index; OR, odds ratio; PA, physical activity; PPS, Physical Program Score; RMSEA, root mean square error approximation SEM, structural equation model; US, United States; VPA, vigorous physical activity; WHO, world health organization.

* Minimum and adequate PA levels of Yu and Ye (75): Minimum MPA: 150 + min/week of MPA and 0 min/week of VPA; Minimum VPA: 75 + min/week of VPA and 0 min/week of MPA; Minimum MVPA: 150 + min/week of VPA combined with MPA; Adequate MPA: 300 + min/week of MPA and 0 min/week of VPA; Adequate VPA: 150 + min/week of VPA and 0 min/week of MPA; Adequate MVPA: 300 + min/week of VPA combined with MPA.

The participants of the studies represented a diverse population in terms of socio-demographic factors known to influence grit and resiliency (e.g., age, gender, socio-cultural, age). There were a total of 37,370 participants (18,614 Male; 16,883 Female) in the included studies with 4 distinct subject populations consisting of students (*n* = 13,657; 7,347 Male; 6,310 Female), healthy adult populations (*n* = 12,382; 3,217 Male; 7,342 Female), athletes (*n* = 5,113; 3,896 Male; 1,167 Female), military populations (*n* = 7,743; 6,364 Male; 1,379 Female) and older adults (defined as 60 + years; *n* = 979; 550 Male; 429 Female). All included both males and females, however one study did not report the sex of the participants (54).

While some studies included exclusively young adults (*n* = 17) (14, 15, 28, 52, 55, 56, 58, 63, 64, 67, 69, 70, 74, 75, 77–79), middle-aged adults (*n* = 2) (62, 76), or older adults (*n* = 3) (46, 65, 71), other studies (*n* = 4) included adults across the age span (15, 16, 51, 72), including 3 of 4 studies in Martin et al. (15) (studies 1, 2, & 4).

The educational backgrounds of participants in the included studies varied, with college students being the focus of a subset (*n* = 13/37 or 35%) of the studies (14, 15, 28, 52, 56, 58, 63, 64, 69, 70, 74, 77–79), whereas *n* = 19/37 (∼51%) of the studies did not report educational level of participants. Only one study (16) reported and controlled for education in their analysis.

The included studies were conducted in 7 different countries including the United States (*n* = 20/37) (14–16, 28, 38, 51, 52, 53, 54, 55, 56, 57, 67, 76, 77, 80, 81), Spain (*n* = 4/37) (46, 69, 59), Australia (*n* = 3/37) (64, 68, 72), China (*n* = 3/37) (63, 74, 75), United Kingdom (*n* = 2/37) (62, 71), Iran (*n* = 2/37) (58, 79), and Turkey (*n* = 1/37) (70).

Less than half (∼45%) of the included studies used only a grit (*n* = 15/37) while ∼57% used a resilience (*n* = 21/37) only survey instrument, and three studies used both grit and resilience survey instruments (Table 2) (14, 76, 77). Grit survey instruments included the 8-item Grit-Short Scale (*n* = 13) (14–16, 28, 38, 53, 55, 57, 58, 76, 77) and the 12-point Grit Scale (*n* = 5) (51, 52, 54, 56). Resilience survey instruments included the 10-item Connor-Davidson Resilience Scale (CD-RISC-10; *n* = 11) (14, 46, 60, 62, 63, 65, 68, 69, 74–76), the Brief Resilience Scale (BRS; *n* = 6) (59, 61, 66, 67, 71, 72), the Psychological Resilience Scale (*n* = 1) (70), the Wagnild & Youngs Psychological Resilience Scale (*n* = 1) (73), the Dispositional Resilience Scale (55), and the Psychological Capital Questionnaire (*n* = 1) (64).

### 3.3 Physical activity adherence

Approximately 83% (*n* = 30/37) of included studies reported a positive association between grit and/or resilience and PA outcomes (Table 2). All extracted studies employed two main analytical approaches: group comparisons or examining relationships between grit or resilience and PA outcomes. Of the studies that performed group comparisons (*n* = 11), 9 reported that participants who engaged in higher levels of PA reported higher levels of grit (*n* = 4) (14, 16, 52, 57) or resilience (*n* = 5) (14, 46, 65, 68, 73) compared to those not engaging in PA. However, Martin et al. (28) reported no significant differences in grit between individuals who met PA guidelines compared to those who did not, while Blanco-Garcia et al. (59) reported no significant difference in resilience between different sport types or levels. Studies (*n* = 10) reported that grit (*n* = 5) (16, 54, 55, 58, 76) and resilience (*n* = 5) (62, 69, 71, 72, 76) were lower for those engaging in lower levels of PA, but still physically active.

Furthermore, 69% of all studies used multivariate regression, or bivariate correlation analyses, while two studies (Flinchbaugh et al., study 1 and 2) (51) used structural equation modeling (SEM). Of these studies, 18 identified a positive relationship between various aspects of PA engagement and grit (*n* = 10) (14–16, 27, 52, 53, 55, 78) or resilience (*n* = 8) (14, 60, 63, 66, 67, 70, 74, 80). This positive relationship was reported in 14 of 16 studies among college students (14, 15, 52, 55, 57, 58, 61, 63, 64, 66, 67, 69, 70, 74, 75). For example, Dunston et al. (14) found that in a sample of US college students, vigorous PA was positively correlated with resilience and perseverance of effort. Moreover, those who met vigorous PA recommendations had higher grit and resilience scores than those not meeting the recommendations (14). The only study that did not report a positive association between grit and PA was Martin et al. (28), which reported a significant relationship between VPA and grit in a non-significant model. Chow and Choi (61) reported no significant relationship between resilience and physical activity.

Multiple studies investigated the impact of grit (*n* = 6) (15, 16, 28) and resilience (*n* = 4) (62, 63, 67, 72) on adherence to PA during the COVID-19 pandemic, which presented new challenges such as gym closures and physical distancing measures that hindered many individuals from maintaining their PA levels. Greater levels of resilience (63, 67) and grit (15, 16) were reported to be positively associated with PA levels in college students (15, 63, 67) and US Adults (15, 16) during the COVID-19 pandemic. Similarly, Australian adults who reported meeting PA guidelines had greater levels of resilience than those who did not meet PA guidelines (72). Moreover, elite endurance athletes, who exhibited higher levels of resilience than amateur athletes, perceived fewer barriers in continuing their training during the pandemic (62). Interestingly, one study found a positive relationship between grit and PA in US college students during the COVID-19 pandemic, however, when dietary and sleep behavior predictors were added to the stepwise regression model, PA was no longer associated with grit (28). In 2 of 4 studies reported in Martin et al. (studies 2 and 4) (15), the authors report that for individuals who have jobs that require them to be physically active, there is no significant association between PA and grit.

### 3.4 Physical activity intensity

A subset of the studies reported on the association between physical activity intensity and grit (*n* = 7) (15, 16, 28, 55) or resilience (*n* = 5) (60, 64, 67, 72, 75), with one study reporting an association between both grit, resilience and PA (14). Of these 13 studies (14–16, 55, 60, 64, 67, 72, 75), 5 reported a positive association between grit, resilience to high levels of PA intensity, specifically vigorous activity (14, 16, 55, 60, 64), while Dunston et al. (28) reported no significant association between VPA and grit when accounting for diet and sleep behavior.

Results for moderate to light PA were conflicting. Dunston et al. (14) report a significant association between vigorous PA, resilience, grit perseverance of effort, and grit consistency of interest. However, moderate PA was correlated with grit perseverance of effort and grit consistency of interest only, while walking time, was not associated with either aspect of grit or resilience (28). These findings are contradictory to those presented by Daniels et al. (55), who report a significant association between grit, as well as the two sub-constructs of grit and VPA, MPA, and walking in a sample of college students. While Dunston et al. (14) reported a significant negative association between grit consistency of interest and VPA and MPA, Daniels et al. (55) report a significant positive association between these outcomes. Further, Lines et al. (64) and Peyer et al. (67) report contradictory findings in that resilience is associated with LPA (64) and MPA (64, 67) in their sample of university students, while Dunston et al. (14) report no significant association between resilience and LPA or MPA.

Conversely, in adults during the COVID-19 pandemic, Totosy de Zepetnek et al. (16), report a significant association between grit and VPA, but with MPA or LPA, while Carriedo and colleagues (60) reported similar results for all components of resilience and VPA, but not MPA or LPA. For studies that combined VPA and MPA and examined MVPA (15, 72) or combined VPA, MPA and LPA to report an overall PA outcome (74) find that MVPA is positively associated with grit (15) and resilience (72), while overall PA is also positively associated with resilience (74).

When examining levels of athletic competitions, athletes competing at higher levels within a sport report higher levels of grit (54, 58) and resilience (62, 68). However, these findings contradict those of Blanco-Garcia et al. (59) who report no differences in resilience among athletes at various levels of play within a sport.

### 3.5 Domains of physical activity

Grit and resilience were assessed across various domains of PA, including recreational activities (55, 62, 76), team and individual sports (54, 58, 59, 62, 78, 79), as well as within military settings (53, 56, 77). Of the 6 studies that assessed levels of competition grit and resilience, five reported that athletes at higher levels of competition reported higher levels of grit (54, 58, 78, 79) and resilience (62) compared to their lower-level (54, 62, 78, 79) or non-athlete peers (58). Shamshirian et al. (58) found that wrestlers showed greater levels of grit compared to a control group of students, although no differences in grit were found between international and national level wrestlers. Furthermore, elite CrossFit athletes scored significantly higher on the 12-item grit scale compared to novice CrossFit athletes (54). In contrast, Blanco-Garcia et al. (59) reported no significant differences in resilience between sport levels or sports category in a study of 1,047 competitive Spanish athletes. However, Blanco-Garcia et al. (59) had no comparator group, as all athletes in their study were participating at a competitive level in Spain and levels were selected based on whether they compete on the national team or not. Another interesting finding was that amongst wheelchair rugby athletes, those with higher levels of grit were the most engaged with their sport (76).

In populations of members of the military, grit and resilience are positively associated with various measures of military fitness tasks when assessed in cadets (53, 56) or during Ranger Training (77); however, grit is not associated with physical activity levels in individuals who are not in training (15). Grit was positively associated with military cadet performance on their physical program score (56), an outcome that assesses instructional coursework, fitness testing and participation in competitive sports and on the strength portions of cadets' fitness tests (77), while resilience is associated with all aspects of cadets' fitness tests. Grit is also positively associated with push-ups and the number of days that Ranger Trainees participate in resistance training and rucking (77).

### 3.6 Socio-Demographic factors

#### 3.6.1 Age

A majority (*n* = 29/37) studies consistently demonstrated positive associations between both grit and resilience with PA across different life stages. Specifically, the research showed a beneficial influence of grit (14, 15, 52, 58, 79, 81) or resilience (14, 63, 67, 69, 70, 74, 75) on PA in young (14, 52, 55, 58, 63, 67, 69, 70, 74, 75, 79) and older adults (46, 65, 73, 71, 80) as well as adults across the lifespan (15, 16, 38, 51, 60, 62, 68, 72, 76). The one exception was Thogersen–Ntoumani et al. (71), who found **no** association between resilience and any PA domain in older UK manual workers. Thus, aside from this outlier, age was not found to systematically alter the positive grit/resilience relationship with PA.

#### 3.6.2 Sex

All studies included both sexes, however, sex differences in the grit, resilience and physical activity outcomes were not an aim of most studies. Peyer and colleagues (67) reported that resilience had a stronger association with PA levels in males than females. Additionally, of the 25 studies that performed regression analyses, 16 studies accounted for sex in their models (14–16, 28, 38, 52, 55, 57, 60, 72, 74, 75, 77). Of these studies, some found an influence of sex (52, 57) or difference between sexes (55, 59, 60, 67), while other studies reported no sex differences (28, 62, 70) on variables of interest (e.g., grit, resilience, PA measures). Thus, the evidence on sex as a moderator is mixed and does not point to a consistent influence of sex on the grit/resilience relationship with PA.

#### 3.6.3 Education

Education level was rarely reported. Over half of studies did not specify participant education, and most samples (e.g., university students, military cadets) implied at least secondary/college education (Table 2). Only one study explicitly controlled for education (16). Totosy de Zepetnek et al. (16) found that higher that higher levels of education and vigorous PA were associated with greater levels of grit. With no other study including education as a moderator its influence on the grit/resilience relationship with PA remains relatively unknown.

#### 3.6.4 Socio-Cultural

The 37 studies were conducted across multiple regions and most were in Western countries (e.g., USA, Spain, UK, France) but there were others in Asia (e.g., China, Iran, Turkey), Australia, and Brazil (Table 2). Despite this socio-cultural diversity, results were remarkably consistent across contexts. For example, U.S., Spanish, Chinese, and other samples all showed that more-active participants reported higher grit or resilience. In one Brazilian longitudinal study (73), older adults who remained regularly active over four years had significantly higher resilience than intermittently active peers. These cross-cultural findings suggest the grit/resilience relationship with PA is robust across settings, although no study formally tested culture or ethnicity as moderators (Table 3).

**Table 3:** Summary of socio-demographic factors and Key findings.

Study	Trait Assessed	Population Age Group	Sex	Education	Culture/Region	PA Outcome(s)	Key Finding
(A) Significant relationships between grit and/or resilience and physical activity outcomes							
Allee et al. (52)	Grit	Young adult (university students)	Both	Not reported	USA	Daily step count	Grit not related to steps until adjusted for age/sex
Benedict et al. (53)	Grit	Adult (military)	Both	Not reported	USA	Weekly strength training frequency; ruck march frequency	Grit showed weak positive correlations with weekly strength training and ruck-march days
Cazayoux & DeBeliso (54)	Grit	Adult	Both	Not reported	USA	CrossFit performance level	Advanced CrossFitters had higher total grit and consistency than novices.
Daniels et al. (55)	Grit	Young adult (university students)	Both	Not reported	USA	MET-min/week	Grit positively predicted total, vigorous, moderate, walking, domestic, and leisure PA.
Flinchbaugh et al. (51) Study 2	Grit	Adult (cyclist athletes)	Both	Not reported	USA	Weekly training volume	Greater weekly training volume was positively related to grit subscales.
Kelly et al. (56)	Grit	Young adult (military)	Both	Not reported	USA	Physical Program Score	Higher total grit and subscales correlated with better physical performance.
Martin et al. (28)	Grit	Young adult	Both	Not reported	USA	MET-min/week	Grit positively associated with vigorous PA.
Martin et al. (15) Study 1	Grit	Adult	Both	Not reported	USA	MVPA	Grit positively predicted MVPA in U.S. adults.
Martin et al. (15) Study 2	Grit	Adult (civilians)	Both	Not reported	USA	MVPA	MVPA predicted higher grit in non-military US adults.
Martin et al. (15) Study 3	Grit	Young adult (university students)	Both	Not reported	USA	MVPA	Grit positively predicted MVPA in university students.
Nothnagle & Knoester (38)	Grit	Adult	Both	Not reported	USA	Childhood/adult sport participation	Grit higher in adults with continual childhood/adult sports participation.
Reed (57)	Grit	Adult (university students and adults)	Both	Not reported	USA	Exercise behavior	Individuals who exercised had higher grit and exercise behavior positively predicted grit.
Shamshirian et al. (58)	Grit	Young adult (athletes and university students)	Male only	Not applicable	Iran	Athletic status	Elite wrestlers scored higher on grit than student controls.
Totosy de Zepetnek et al. (16)	Grit	Adult	Both	Controlled for education	USA	VPA/MPA/LPA	Grit positively related to VPA and participants with ≥300 min/wk PA had higher grit.
Carriedo et al. (60)	Resilience	Adult	Both	Not reported	Spain	VPA/MPA/LPA	VPA/MPA were positively correlated with locus of control, self-efficacy, and optimism resilience subscales.
Harman et al. (62)	Resilience	Adult	Both	Not reported	France/UK	Weekly training volume; athletic level	Higher resilience was associated with higher athletic level and fewer perceived training barriers.
Li et al. (63)	Resilience	Young adult	Both	Not reported	China	Exercise adherence	Exercise behavior was strongly correlated with resilience.
Lines et al. (64)	Resilience	Young adult (university students)	Both	Not reported	Australia	LPA/MPA/VPA	VPA, MPA, and LPA showed positive correlations with resilience at various time points.
Martínez-Moreno et al. (46)	Resilience	Older adult	Both	Not reported	Spain	PA engagement	Older adults who exercised had significantly higher resilience than inactive peers.
Martínez-Moreno et al. (65)	Resilience	Older adult	Both	Not reported	Spain	PA engagement	Physically active older adults had higher resilience.
Ozkara et al. (66)	Resilience	Young adult (university students)	Both	Not reported	Turkey	Childhood PA level	Childhood PA level positively predicted adult resilience.
Peyer et al. (67)	Resilience	Young adult (university students)	Both	Not reported	USA	Strength training & moderate exercise frequency	Resilience was positively related to strength training (both sexes) and to moderate exercise in men; men meeting guidelines had highest resilience.
Roebuck et al. (68)	Resilience	Adult	Both	Not reported	Australia	Running distance/time; strength training frequency	Ultramarathoners had significantly higher resilience than non-runners.
San Román-Mata et al. (69)	Resilience	Young adult (university students)	Both	Not reported	Spain	Meeting PA guidelines	Meeting weekly PA guidelines was associated with higher total resilience and all subscales.
Seçer and Çakmak Yıldızhan (70)	Resilience	Young adult (university students)	Both	Not reported	Turkey	MET-min/week	PA significantly predicted resilience in Turkish students.
To et al. (72)	Resilience	Adult	Both	Not reported	Australia	Meeting MVPA guidelines	Meeting MVPA guidelines predicted higher resilience in both longitudinal and cross-sectional samples.
Wermelinger Ávila et al. (73)	Resilience	Older adult	Both	Not reported	Brazil	Regular vs. intermittent PA over 4 years	Older adults who remained regularly active for 4 years had significantly higher resilience than intermittently active peers.
Xu et al. (74)	Resilience	Young adult (university students)	Both	Not reported	China	MVPA/LPA	PA positively predicted resilience in Chinese university students.
Yu and Ye (75)	Resilience	Young adult (university students)	Both	Not reported	China	Meeting MPA/VPA/MVPA guidelines	Higher resilience increased odds of meeting MVPA recommendations.
Atkinson and Martin (76)	Grit and Resilience	Adult	Both	Not reported	USA	Sport engagement	In wheelchair rugby athletes, both grit and resilience predicted greater sport engagement.
Dunston et al. (14)	Grit and Resilience	Young adult (university students)	Both	Not reported	USA	VPA	VPA positively predicted resilience and perseverance of effort. but negatively predicted consistency of interest.
Schaefer et al. (77)	Grit and Resilience	Young adult (military)	Both	Not reported	USA	Military Movement Course scores	Both grit and resilience predicted overall military performance; resilience predicted most individual events, whereas grit predicted only the strength event.
(B) Non-significant and negative relationships between grit and/or resilience and physical activity outcomes							
Benedict et al. (53)	Grit	Adult (military)	Both	Not reported	USA	Months of strength training prior to US Army ranger school	Grit not associated with months of strength training prior to entering US Army ranger school.
Flinchbaugh et al. (51) Study 1	Grit	Adult (working)	Both	Not reported	USA	Weekly PA volume	PA negatively predicted consistency-of-interest but no association with perseverance of effort.
Martin et al. (15) Study 2	Grit	Adult (active-duty military and veterans)	Both	Not reported	USA	MVPA	MVPA was not associated with grit in active-duty or veterans.
Martin et al. (15) Study 4	Grit	Adult (performing artists)	Both	Not reported	USA	MVPA	Grit was *not* associated with MVPA in performing artists.
Blanco-García et al. (59)	Resilience	Young adult (athletes)	Both	Not reported	Spain	Sport participation	No significant resilience differences by sport type or level.
Chow and Choi (61)	Resilience	Young adult (university students)	Both	Not reported	China	Leisure PA	No significant correlation between leisure PA and resilience.
Thogersen-Ntoumani et al. (71)	Resilience	Older adult	Both	Not reported	UK	Work/leisure/sports PA	No significant associations between resilience and PA were found in older manual workers.
Dunston et al. (14)	Grit and Resilience	Young adult (university students)	Both	Not reported	USA	VPA	VPA negatively predicted consistency of interest.

LPA, light physical activity; MET, metabolic equivalent; MPA, moderate physical activity; MVPA, moderate to vigorous physical activity; PA, physical activity; VPA, vigorous physical activity.

## 4 Discussion

This systematic review synthesized the current knowledge from studies examining the relationship between grit, resilience, and PA. The overall body of research suggests that both grit and resilience positively influence physical activity engagement, intensity of physical performed, and gritty or resilient individuals are more likely to engage in high level competition. While most of the literature was moderate-to-high quality, many of the studies were cross-sectional in nature, which inherently limits extrapolating causality (50). The three studies that used longitudinal designs reported that individuals who were gritty and/or resilient were more physically active across most of the time points when data was collected (64, 72, 73). However, grit and resilience are constructs that may take years to modify, thus interventional trials may not be feasible. Further, evidence suggests that greater levels of fitness, an outcome of regular engagement in PA, may make individuals more resilient to nonphysical stressors (82). This highlights difficulty in addressing the directionality of the relationship between the grit, resilience, and PA. Potential solutions to this challenge may be to use interventions meant to increase PA to determine whether those resulted in an increase in grit or resilience, or to use interventions such as growth mindset that have been known to increase grit (83) to determine whether increased grit resulted in an increase in PA participation. Another challenge with the studies presented in this systematic review is that most of the studies used self-reported measures of PA, such as the IPAQ, which previous work suggests has inherent self-report bias (84). However, the present review presents compelling evidence that individuals who are gritty and/or resilient are more likely to engage in PA, specifically higher intensity PA, and may be more likely to participate at higher levels in their sports.

An important theme to emerge is that the findings indicated that regardless of age, sex, culture or education level, grit and resilience support a variety of PA outcomes. Not only were there positive associations between grit, resilience, and PA participation and adherence, but group comparisons in studies also consistently reported that individuals engaging in higher levels of PA demonstrated greater levels of grit or resilience compared to those not engaging in PA or those with lower PA levels. Leading a physically active lifestyle necessitates consistent effort and determination to overcome barriers (5), which can change as one ages (85–88). Grit represents the ability to adhere to and persevere in the pursuit of long-term goals (20, 29), which is essential for maintaining PA engagement over an extended period. Concomitantly, resilience emerges as an asset for overcoming setbacks and navigating barriers to engage in PA, throughout the lifespan (5).

While we are unaware of evidence that may explain why individuals who are gritty or resilient are more likely to participate in PA, we hypothesize that these individuals may be more likely to set goals and stick to these goals despite setbacks. In other contexts, the personality traits embodied in grit and resilience have been found advantageous. The ability to overcome adversity and setbacks regarding health, such as injuries, acute or chronic illness, is beneficial for improved PA-related outcomes (89), recovery time (90, 91), and overall quality life (92). For example, Traino and colleagues reported in college students diagnosed with a chronic medical conditions those with higher levels of grit were less likely to be discouraged by setbacks and may be more likely to adhere to treatment plans, engage in necessary rehabilitation activities, and follow medical plans (93). Another potential explanation may be that since grit and resilience, which are both part of the conscientiousness family of traits (21), individuals who are gritty/resilient are more likely to adopt behaviors that that they view as long-term investments in one's health (22).

A second important theme from the literature was the association with grit and resilience with higher intensity (e.g., vigorous) PA considering that health benefits tend to be the greatest with higher intensity levels of PA (1, 94, 95). There are several plausible explanations as to why grit and resilience are associated with participation in higher intensity PA. Higher intensity exercise is often more challenging, requiring adaptability and a willingness to face discomfort (96). Resilient individuals thrive in challenging environments, viewing them as opportunities for growth and improvement (97), and they may view performing high intensity physical activity as an opportunity to grow and improve. Individuals with grit and resilience may possess an understanding of the long-term health benefits associated with higher intensity PA. As such, these individuals may prioritize their health goals, recognizing that engaging in more intense PA is linked to improved cardiovascular health, metabolic benefits, and overall well-being. Considering that grit is associated with conscientiousness (21) these findings may be an extension of the “invest and accrue” model (22) to PA where gritty and resilient individuals choose to invest in physical health by engaging in higher intensity PA for future health benefits.

The results pertaining to higher intensity PA can also be understood through established motivational frameworks. According to Self-Determination Theory, sustained engagement in demanding behaviors such as vigorous PA is driven by autonomous motivation, which is motivation stemming from intrinsic enjoyment or the perceived personal value of the activity (98). Individuals with higher levels of grit may be more likely to internalize PA as integral to their identity or long-term goals, thereby exercising for reasons aligned with intrinsic or identified regulation. This interpretation is supported by recent evidence indicating that grit is positively associated with self-efficacy and autonomous motivation for exercise, both of which predict an individual's readiness to initiate and maintain PA (99). In essence, gritty individuals may perceive high-intensity exercise as personally meaningful or rewarding, enabling them to persist despite physical discomfort. Achievement Goal Theory offers a complementary perspective, suggesting that individuals high in grit and resilience are more likely to adopt mastery-oriented goals, focused on personal growth and sustained effort, rather than ego-oriented goals that emphasize outperforming others (100). A mastery orientation has been linked to greater intrinsic motivation and long-term persistence in sport settings (101), which leads to another theme identified in the reviewed studies.

The third theme that emerged was the role of grit and resilience in facilitating higher levels of success within sport and military environments. However, due to study design it is unclear whether reaching these levels of success in sports and military results in increased grit or whether grit is responsible for helping these individuals reach these higher levels. Literature suggests that the attributes of grit, such as perseverance, passion, and sustained effort, are vital for success in competitive arenas (19, 20). Individuals with higher levels of grit are goal-driven, enabling them to persist in the face of challenges and setbacks (102). Moreover, grit fosters a growth mindset, instilling the belief that abilities and performance can be developed through effort and practice (103). This mindset encourages individuals to embrace challenges, seek feedback, and continuously learn and enhance their skills (104). Furthermore, the perseverance aspect of grit may drive athletes to invest dedicated time and effort into deliberate practice, which is essential for skill development and mastery (105). Resilience supports overcoming obstacles which may result in an increased commitment to their training (106). However, it is conceivable that by participating in higher levels of sports and military training individuals may have increased their resilience and/or grit over time. A study from our review suggests that individuals who participated in childhood sport, and continued childhood sport participation were grittier (38). The cross-sectional nature of that study could suggest that either gritty individuals were the ones who continued to participate in sports or that continued sport participation during childhood resulted in higher levels of grit as adults when the participants of that study completed the grit survey.

### 4.1 Limitations

Several limitations related to the search and the methodology of included studies should be considered. Regarding the search process, the review included studies examining sport performance and competition levels. It is important to clarify that these domains were included as proxies for physical activity in certain populations. Sport performance and competition levels often require sustained physical engagement and training that align with or exceed recommended levels of moderate-to-vigorous physical activity. These contexts were considered relevant to this review as they provide insight into PA behaviors in populations where activity is structured around sport-specific demands. However, the authors acknowledge the distinction between direct measures of PA and sport-related outcomes, and this review aims to critically evaluate these distinctions while synthesizing the broader relationships between grit, resilience, and PA outcomes. The type of review conducted could be viewed as a limitation. Ultimately, a systematic review was chosen to provide a structured and transparent approach to synthesize the evidence. As the authors had prior knowledge of the general body of literature it was discussed that a narrative or rapid review would not adequately capture the breadth and depth of the research, while the heterogeneity of the included studies made a meta-analysis impractical.

A general shortcoming of the included studies the overall body of literature did not account for participants' level of interest or goals regarding PA. Grit is a goal-driven trait (25) and future studies assessing PA should consider incorporating a question with the grit survey that captures whether participants engage in PA out of necessity or have specific goals related to PA. Since grit is characterized by passion and perseverance towards long-term goals, individuals whose goals are misaligned with PA participation may participate in lower levels of PA. This was inadequately addressed in the reviewed studies, which limits the interpretation of the findings by introducing potential variations among individuals driven by divergent PA motives. Considering that grit is associated with sustained effort towards achieving long-term goals, while resilience is a broader construct that encompasses the ability to cope with and rebound from various challenges and adversities (31), these traits may be of unique value in different circumstances to support PA outcomes. A second limitation is that, by including both grit and resilience in the present review, we adopted a broad approach to synthesizing the literature on these related constructs. As a result, we did not conduct a more detailed analysis of grit's subcomponents (i.e., consistency of interest and perseverance of effort). Future research should address this gap to better understand how individual components of grit may differentially influence physical activity engagement. Third, a potential source of bias in the systematic review may stem from publication bias, where studies with statistically significant results are favored for publication (107). Another potential limitation is that most studies used self-report PA data, which has been shown to be inherently biased (84). To address potential publication bias in future research, investigators are encouraged to pre-register study protocols and hypotheses, which promotes transparency and helps prevent selective reporting (108). Additionally, researchers can be encourage to publish null findings as they are important to disseminate to add to the body of knowledge on a topic (109). Although sociodemographic and cultural factors likely influence the development and expression of grit and resilience (43, 45), the included studies did not provide sufficient evidence to determine whether the relationships between these traits and physical activity differ across cultural contexts. Most studies were conducted in Western, individualist societies, limiting the generalizability of findings. Future research should investigate whether cultural orientation (e.g., collectivist vs. individualist values) moderates the associations between grit, resilience, and PA behaviors.

A final, and arguably primary, limitation was that while the current body of research suggests a positive association between grit, resilience, and PA behaviors, most of the included studies were cross-sectional, limiting the ability to infer causality or directionality. As a result, it remains unclear whether higher grit or resilience promotes greater PA engagement, or whether regular PA participation contributes to the development of these traits. Future research should employ longitudinal study designs to assess how grit and resilience may change over time in relation to PA behaviors. Additionally, randomized controlled trials that target either the enhancement of grit/resilience (e.g., through mindset training, goal-setting programs) or promote PA (e.g., structured exercise interventions) could help clarify causal pathways. For example, interventions aimed at increasing PA could assess whether sustained engagement leads to improvements in grit or resilience over time. Conversely, interventions focused on enhancing psychological traits could measure their downstream effects on PA adherence and intensity. Such research designs would provide stronger evidence regarding the modifiability of these traits and their potential as intervention targets to promote long-term PA outcomes.

## 5 Conclusion

In conclusion, this systematic review provides valuable insights into the relationship between grit and resilience with PA. The findings of this review contribute to our understanding of how grit and resilience may be associated with PA participation and adherence, which are important for health and wellbeing across the lifespan. It highlights the importance of considering personality traits, encompassed in grit and resilience, as factors in promoting and sustaining PA behaviors. Future research should further explore the relationship between grit, PA, and motivation, as motivation plays a crucial role in initiating and maintaining exercise habits. From a practical standpoint, incorporating grit and resilience assessments into the initial screening process in real-world settings, such as athletic teams, community fitness programs, or clinical exercise interventions, could help identify individuals who may be at higher risk for poor adherence or dropout. These assessments could then inform individualized strategies, such as incorporating psychological skills training, goal-setting, or structured social support, to enhance commitment and persistence. In clinical populations, such assessments may also help clinicians tailor rehabilitation or health promotion programs to better address patient needs and increase long-term engagement in PA.

## Data Availability

The original contributions presented in the study are included in the article/Supplementary Material, further inquiries can be directed to the corresponding author/s.
